# Self‐Stratification of Polymer Films by Strong Coupling Between Molecular Absorbers and Infrared Grating Modes

**DOI:** 10.1002/nap2.70354

**Published:** 2026-11-06

**Authors:** Yunus Çayır, Md Faysal Hossain, Wonmi Ahn

**Affiliations:** ^1^ UNAM—National Nanotechnology Research Center and Institute of Materials Science and Nanotechnology Bilkent University Ankara Türkiye

**Keywords:** dark states, deep metal grating, molecular vibrations, polaritonic states, uncoupled states, vacuum Rabi splitting, vibrational strong coupling

## Abstract

Strong coupling between deep metal grating modes and molecular vibrations provides a powerful route to sculpt the optical response of chemically uniform polymer films. Here, we integrate poly(methyl methacrylate) (PMMA) with a deep silver (Ag) grating to realize vibrational strong coupling (VSC) to the C=O stretching mode, and use full‐wave simulations to disentangle polaritonic, dark, and uncoupled molecular manifolds. We show that the evanescent field of the grating defines an effective mode volume within which PMMA molecules are hybridized into upper and lower vibrational polaritons, while molecules outside this region retain bulk‐like absorption at the bare vibrational frequency. Exploiting this binary optical behavior, we demonstrate a VSC‐based open‐cavity solvent‐diffusion sensor. Ethanol‐induced swelling of PMMA modulates the polaritonic dispersion and absorption at the bare molecular frequency in a depth‐dependent fashion, enabling us to detect the arrival of the solvent front at the strongly coupled interface. Our results establish deep metal gratings coupled to molecular films as a versatile platform for engineering spatially selective strong coupling, with prospects for advanced photonic sensing and spatial control of light–matter coupling in soft materials.

## Graphical abstract

Strong coupling between an infrared deep Ag grating mode and the C=O stretch of poly(methyl methacrylate) (PMMA) induces effective self‐stratification in an otherwise chemically uniform film. Using rigorous coupled‐wave analysis, we calculate optical spectra and electric‐field distributions, showing that the evanescent grating field partitions the PMMA into distinct optical regions, producing a self‐stratified response unattainable by refractive‐index engineering alone.

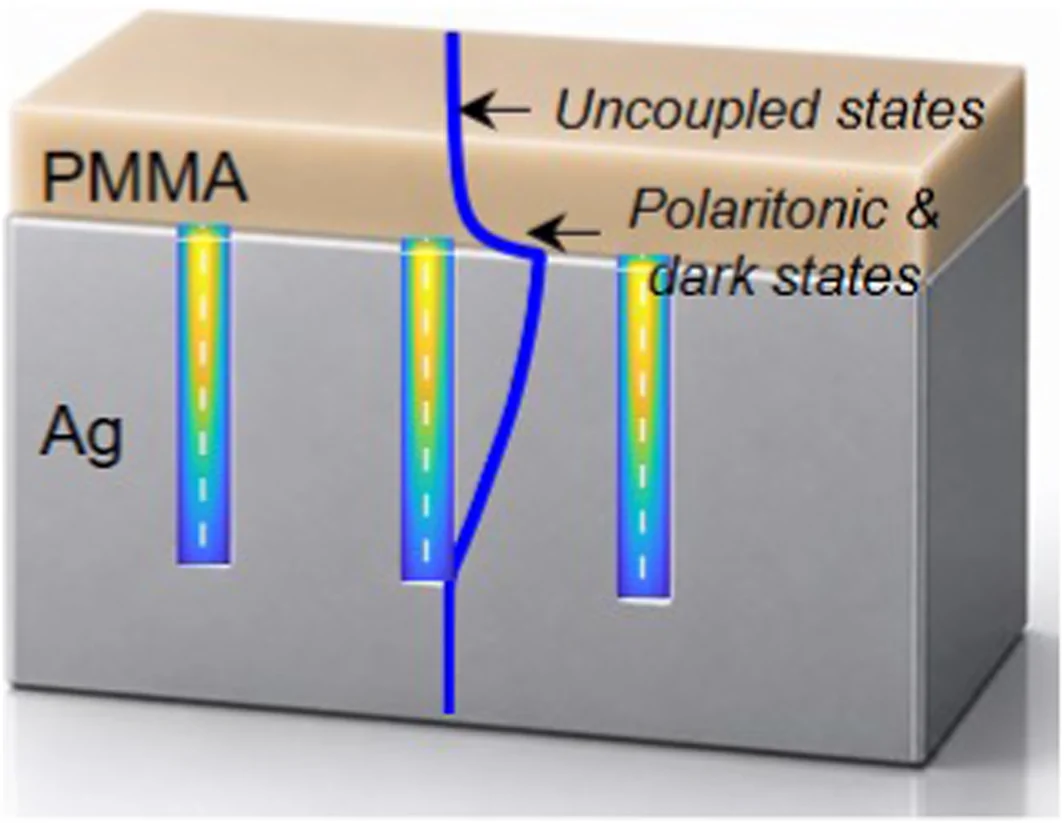

## 1 Introduction

Molecular systems can exhibit fundamentally altered physical properties when their vibrational modes are strongly coupled to a resonant optical cavity [1, 2]. Vibrational strong coupling (VSC) is established when the light–matter interaction strength exceeds the dissipative rates of both the molecular and photonic modes, driving the system into the strong coupling regime [3, 4]. In this regime, hybrid light–matter states, known as vibrational polaritons, emerge and are spectrally resolved as upper (UP) and lower polariton (LP) branches separated by the Rabi splitting ($\Omega_{R}$). The magnitude of the Rabi splitting scales with the square root of the number of molecules N coupled to the cavity mode, reflecting the collective nature of the coupling [5]. Molecules participating in polaritonic states also inherit characteristics from the photonic modes, such as reduced effective mass and angle‐dependent dispersion [6]. As a result, vibrational polaritonic systems have been shown to reshape material properties, in particular, modifying ground‐state chemical reactivity [7, 8]. Despite these advances, comparably little attention has been given to self‐stratification of polymer films by VSC.

In addition to the polaritonic branches, strong light–matter coupling forms a manifold of *dark* states [9], comprising N − 1 nearly degenerate states for an ensemble of N. These states are orthogonal to the bright polaritonic states and, therefore, do not couple to the cavity field, rendering them optically dark [10]. While dark states retain predominantly molecular character with negligible photonic component, they can nevertheless inherit the delocalized character of the polaritonic states in confined photonic environments [11]. An increasing body of evidence indicates that dark states play a nontrivial and often dominant role in strongly coupled systems. In particular, they have been shown to facilitate energy transport [12, 13], govern relaxation dynamics [14, 15], and extend polariton propagation lifetimes [16], in excitonic strong coupling systems, and contribute to modifications of chemical reactivity in VSC systems [17, 18]. These findings challenge the conventional view that assigns functional relevance primarily to bright polaritonic states.

A distinct but frequently overlooked manifold arises from molecules located outside the cavity mode volume, which remain entirely *uncoupled* from the cavity field. In fact, dark states and uncoupled states can appear spectrally similar, as both are centered near the bare molecular resonance and exhibit negligible dispersion upon cavity tuning. However, their physical origins are fundamentally different. Dark states are an intrinsic consequence of collective strong coupling and coexist with the polaritonic states, whereas uncoupled states do not participate in hybridization and remain unaffected by the cavity field [19]. As the collective coupling strength increases, the dark states become more pronounced at the molecular frequency as the bright state hybridizes strongly and splits into two polaritonic states [20]. In contrast, uncoupled states retain a cavity‐independent spectral response and population, providing a useful internal reference for disentangling true strong‐coupling from bulk effects [21].

Despite their fundamental importance, most strong coupling studies have focused on polaritonic splitting and dynamics, while largely overlooking the identification and characterization of dark and uncoupled states. In fact, direct observation of these states is challenging in conventional Fabry‐Pérot (FP) cavities. High‐finesse cavities support spatially uniform optical fields that preserve dark state orthogonality and minimize disorder‐induced mixing, thereby limiting their optical visibility. Nonetheless, experimental evidence of dark and/or uncoupled states has emerged in FP systems, typically as additional absorption [22] and emission features [23, 24], especially at high molecular densities where intermolecular interactions break symmetry and relax selection rules [25]. Advanced nonlinear spectroscopic techniques, including pump‐probe [26, 27] and two‐dimensional infrared spectroscopy [28, 29], have revealed dark state populations and dynamics even under high‐finesse cavity conditions. Beyond FP cavities, other photonic structures such as plasmonic slit arrays [30], cavities incorporating two‐dimensional materials [31], and open cavity structures including distributed Bragg reflector cavities [32, 33], and holey metal surfaces [34], provide platforms that break orthogonality constraints, making dark and/or uncoupled states optically accessible. Still, comprehensive studies that simultaneously describe polaritonic, dark, and uncoupled states within a single unified strong‐coupling system remain scarce, particularly in the context of VSC. Establishing such a platform would not only clarify their respective roles but also enable systematic control over their interplay, with direct implications for polaritonic chemistry [7], polaritonic quantum processing [35, 36, 37], and polymer photonics, including phenomena such as cavity‐induced self‐stratification.

Self‐stratifying polymer coatings generally refer to the formation of multilayer structures through a single‐step application of one coating composition, while generating surface properties that are different from those of the bulk [38, 39]. Owing to their simplicity and enhanced durability achieved by avoiding interlayer incompatibility, self‐stratifying polymer coatings have been used to improve the mechanical strength [40], surface hydrophobicity [41], and electrical insulation of surfaces [42], as well as to introduce advanced functionalities such as antimicrobial [43], antifouling [44], and self‐healing properties [45]. In general, self‐stratified polymer coatings are prepared through phase separation within polymer assemblies by solvent evaporation, interpolymer reactions, surface tension gradients, and kinetic control [46, 47, 48]. However, such chemically‐driven processes are limited to specific polymer systems, and their effectiveness depends on factors such as solvent polarity and viscosity, polymer molecular weight, glass transition temperature, and intramolecular interactions [49, 50, 51].

In this study, we demonstrate the simultaneous formation and spatial coexistence of polaritonic, dark, and uncoupled states in a single platform, and establish VSC as a mechanism for inducing self‐stratification in polymer films. Our system represents a deep silver (Ag) grating strongly coupled to the carbonyl C=O stretching vibration of poly(methyl methacrylate) (PMMA) at 1730 cm^−1^. Under TM‐polarized excitation, deep Ag gratings with grating heights of 0.5–1.1 μm can support magnetic‐polariton resonances associated with antiparallel currents along the metallic trench walls and localized magnetic fields within the trench region [52, 53, 54]. In our previous work, simulated electric‐ and magnetic‐field distributions, together with electric‐field vector maps, revealed circulating electric fields in the trench region and antiparallel currents along the trench sidewalls, consistent with the magnetic polaritons [52]. In addition to magnetic polariton modes, deep Ag gratings can also support surface plasmon modes. In our structure, the surface plasmon contribution is indicated by the pronounced angle dependence of the optical response [52], whereas the localized magnetic polariton contribution is comparably angle insensitive [55]. The relative contributions of the magnetic polariton and surface plasmon modes, and thus their hybridization, depend strongly on the grating geometry, particularly the grating height [52].

As an open cavity platform, the deep Ag grating provides spatial access to the cavity field, allowing systematic investigation of how the number of coupled molecules N and their distance from the optical mode governs light–matter coupling. Within the evanescent decay length of the optical field, the PMMA‐grating system shows clear $\Omega_{R}$ that scales with $\sqrt{N}$, accompanied by a small but non‐zero residual absorption at the bare molecular frequency ($A^{{1730\;\text{cm}}^{-1}}$). In this near‐field region, strong coupling is spatially confined, and both polaritonic and dark‐state manifolds are established. Beyond the decay length, the system progressively departs from collective strong‐coupling behavior. The $\Omega_{R}$ deviates from the expected $\sqrt{N}$ scaling, while the $A^{{1730\;\text{cm}}^{-1}}$ intensity increases and eventually saturates, indicating the regions of the uncoupled states. Therefore, although the polymer film is chemically homogeneous, its molecules can exhibit distinct optical behavior depending on their spatial overlap with the confined optical mode.

Rather than treating the uncoupled molecular contribution as a consequence of spatially nonuniform field distribution alone [56, 57, 58], we systematically vary the PMMA overlayer thickness and track the evolution of $\Omega_{R}$, the absorption at the bare molecular frequency, and the evanescent field profile. This allows us to resolve a depth‐dependent transition from a strongly coupled region near the deep Ag grating to a bulk‐like uncoupled region farther from the grating. Utilizing a binary optical response of the coupled polymer film, we demonstrate solvent‐diffusion sensing enabled by VSC‐induced self‐stratification of a polymer film. The ability to design molecular layers with distinct photonic and material characteristics introduces new possibilities for all‐optical self‐stratification, with potential applications in light‐tunable membrane transport, stratified functional coatings, and polariton‐enabled interfacial photonics and chemistry.

## 2 Results and Discussion

### 2.1 Vibrational Strong Coupling With a PMMA‐Integrated Deep Ag Grating

We used rigorous coupled‐wave analysis (RCWA) simulations, which provide full‐wave solutions to Maxwell's equations for periodic multilayer structures. Unlike simple analytical models, RCWA captures the collective optical response of the system, revealing bright polaritons, dark states, and uncoupled states, as each mode emerges as an eigenstate of the coupled light–matter system [59]. The RCWA has been widely applied to model spectral responses and field distributions of metal gratings [60, 61], slit arrays [62], and chiral metasurfaces [63], demonstrating high accuracy for simulating layered and periodic structures in polaritonic studies [64, 65].

The geometrical parameters of the deep Ag grating were first optimized through a series of RCWA simulations to spectrally align the grating resonance frequency with the C=O stretching mode of PMMA at 1730 cm^−1^. The optimized structure features a grating period (*P*) of 3 μm, a trench gap (*b*) of 50 nm, and a bottom Ag film thickness of 50 nm. Increasing *b* from 50 to 200 nm increases the full width at half maximum (FWHM) of the absorption resonance (Supporting Information S1: Figure S1), indicating the modified electromagnetic confinement within the trench. Varying *b* also shifts the grating‐mode resonance and its spatial overlap with PMMA. The grating height (*h*) was varied between 0.5 and 1.1 μm to investigate *h*‐dependent absorption near the bare molecular resonance. In addition, the thickness of the top PMMA layer ($t_{\text{top}}$) was varied from 0 to 4181 nm to determine the critical distances separating regions between the bright polaritonic, dark, and uncoupled states.

Although the geometric parameters considered were used for the present numerical study, fabrication of deep Ag gratings with such a high aspect ratio remains experimentally challenging. Recent fabrication efforts have demonstrated deep metal grating structures with grating heights of approximately 1.0–2.0 μm [66, 67, 68]. However, after post‐etch processing steps, the trench widths typically remain in the range of 200–300 nm. The difference between the simulated and currently attainable geometries indicates that the 50 nm trench width may be regarded as an optimized numerical design parameter rather than a strict geometrical requirement. In addition, the fully PMMA‐filled 50 nm trench gap is a modeling assumption used in the RCWA calculations and has not been experimentally demonstrated in the present work. In parallel with the present numerical study based on RCWA simulations, our ongoing fabrication efforts are directed toward reducing the trench width while maintaining the required grating depth and structural integrity.

Figure 1 shows absorption intensity maps of (a) a bare deep Ag grating without PMMA, (b) a bare PMMA layer with the $t_{\text{top}}$ varied from 0 to 4181 nm, and (c) a deep Ag grating integrated with PMMA both inside the trench gaps and on the grating top surface, with $t_{\text{top}}$ of 55 nm. A single absorption peak is observed for the bare deep Ag grating within the spectral range from 1400 to 2800 cm^−1^, which red‐shifts as *h* is increased from 0.5 to 1.1 μm (Figure 1a). This resonance arises from the hybridization of magnetic polariton and surface plasmon modes supported by the deep Ag grating [52]. The FWHM of the uncoupled grating mode ($\Gamma_{g}$) is obtained as 142.1 cm^−1^ at *h* = 0.67 μm. On the other hand, the bare PMMA layer shows an *h*‐independent absorption peak corresponding to the PMMA C=O stretching mode at 1730 cm^−1^, with $\Gamma_{\text{PMMA}}$ of 26.8 cm^−1^ for a 55 nm‐thick PMMA layer (Figure 1b). Finally, when PMMA is integrated with the deep Ag grating, VSC manifests itself as a clear anti‐crossing in the *h*‐dependent absorption intensity map (Figure 1c). As the grating resonance is tuned via *h*, the single absorption peak splits into the UP and LP branches relative to the C=O vibrational mode (horizontal dotted line at 1730 cm^−1^). The $\Omega_{R}$ is obtained as 125.7 cm^−1^ at *h* = 0.67 μm, which exceeds the average of $\Gamma_{g}$ and $\Gamma_{\text{PMMA}}$, thereby satisfying the criterion for strong coupling [69].

**FIGURE 1:**
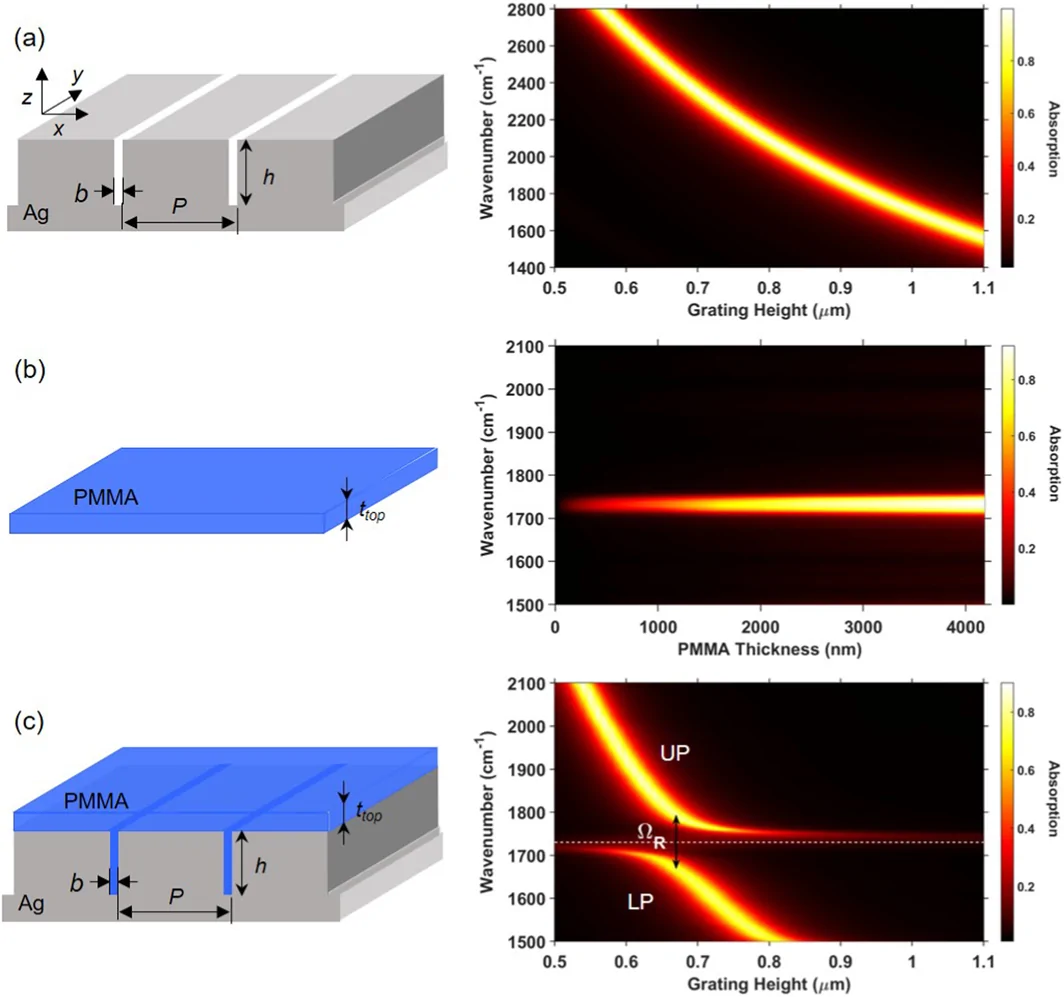
Vibrational strong coupling in a deep Ag grating integrated with PMMA. RCWA‐simulated absorption intensity maps of (a) a bare deep Ag grating without PMMA, with structural parameters *P* = 3 μm, *b* = 50 nm, bottom Ag film thickness of 50 nm, and *h* varied from 0.5 to 1.1 μm, (b) a bare PMMA layer with thickness ($t_{\text{top}}$) varied from 0 to 4181 nm, and (c) a deep Ag grating integrated with PMMA both within the trench gaps and on top of the grating, with $t_{\text{top}}$ of 55 nm. A clear anti‐crossing is observed in (c), where the UP and LP branches form around the C=O resonance of PMMA at 1730 cm^−1^ (horizontal dotted line), yielding a Rabi splitting $\Omega_{R}$ of 125.7 cm^−1^ at *h* = 0.67 μm.

We found that filling the trench gaps with PMMA is crucial for achieving strong coupling. When the trench gaps are left empty (i.e., air‐filled grating), the maximum $\Omega_{R}$ reaches only 79.1 cm^−1^, even at the largest $t_{\text{top}}$ of 4181 nm (Supporting Information S1: Figure S2). This occurs because the strongest electric (*E*) fields are predominantly confined within the trench gaps (as discussed below) and therefore do not contribute effectively to the overall coupling strength unless they partially overlap with PMMA in the gaps. Consequently, the $\Omega_{R}$ value of the air‐filled grating is only 62.5% of that obtained for the PMMA‐filled grating and does not satisfy the strong coupling criterion. In addition, partially filling the trench gaps with PMMA without a top PMMA layer does not readily drive the system into the strong coupling regime either (Supporting Information S1: Figure S3). A filled‐gap PMMA thickness ($t_{\text{gap}}$) of approximately 500 nm is required to reach the strong coupling regime, and even then, coupling occurs at a larger *h* of 0.86 μm. Such a configuration imposes stricter nanofabrication requirements and makes selective filling of only the trench gaps with PMMA experimentally challenging. Therefore, we chose a deep Ag grating structure with PMMA both in the trench gaps and as a top layer, enabling strong coupling while maintaining precise control over the top PMMA thickness via spin coating.

### 2.2 Formation of Polaritonic, Dark, and Uncoupled States

To distinguish between polaritonic, dark, and uncoupled states, we analyzed the linear absorption, reflection, and transmission spectra of the PMMA‐integrated deep Ag grating, focusing on spectral splitting and the residual intensity at the original molecular resonance. First of all, the absorption, reflection, and transmission spectra showed the UP and LP branches, resulting in a constant $\Omega_{R}$ value of 125.7 cm^−1^ (Figure 2). Due to the thick Ag layer in the grating, transmission remains very low across the spectral range, whereas both absorption and reflection exhibit high intensities. At the C=O stretching frequency of PMMA, the transmission is nearly zero, whereas the absorption spectrum retains a residual intensity ($A^{{1730\text{cm}}^{-1}}$) of approximately 15%, independent of *h* (Figure 2a). For a thin top PMMA layer used ($t_{\text{top}}$ of 55 nm), this residual absorption intensity at the molecular resonance is attributed to *dark* states arising from molecules that are incoherently coupled to the cavity field [70]. We emphasize that a similar residual absorption of 15% is still observed even in the absence of a top PMMA layer when the trench gaps are completely filled with PMMA (Supporting Information S1: Figure S4). This rules out contributions from uncoupled states originating from excess molecules physically located outside the cavity mode volume. However, the absence of an appreciable increase in the spectral feature near the bare molecular vibration upon adding a 55 nm‐thick PMMA overlayer can be attributed to the substantially weaker effective field overlap of the overlayer, relative to the PMMA within the trench gaps, as will be shown below. The residual absorption at the molecular resonance can also be used to estimate the fraction of strongly coupled molecules in the cavity [71]. Based on the absorption spectra shown in Figure 2a, approximately 85% of the PMMA molecules are strongly coupled to form polaritonic states, while the remaining 15% contribute to dark states. However, the intensity of the residual spectral feature near the bare C=O vibrational frequency should not be interpreted as a direct measure of the number of dark states. Rather, this feature may include contributions from dark molecular states that become weakly visible because of disorder or loss, together with contributions from weakly coupled and effectively uncoupled PMMA molecules.

**FIGURE 2:**
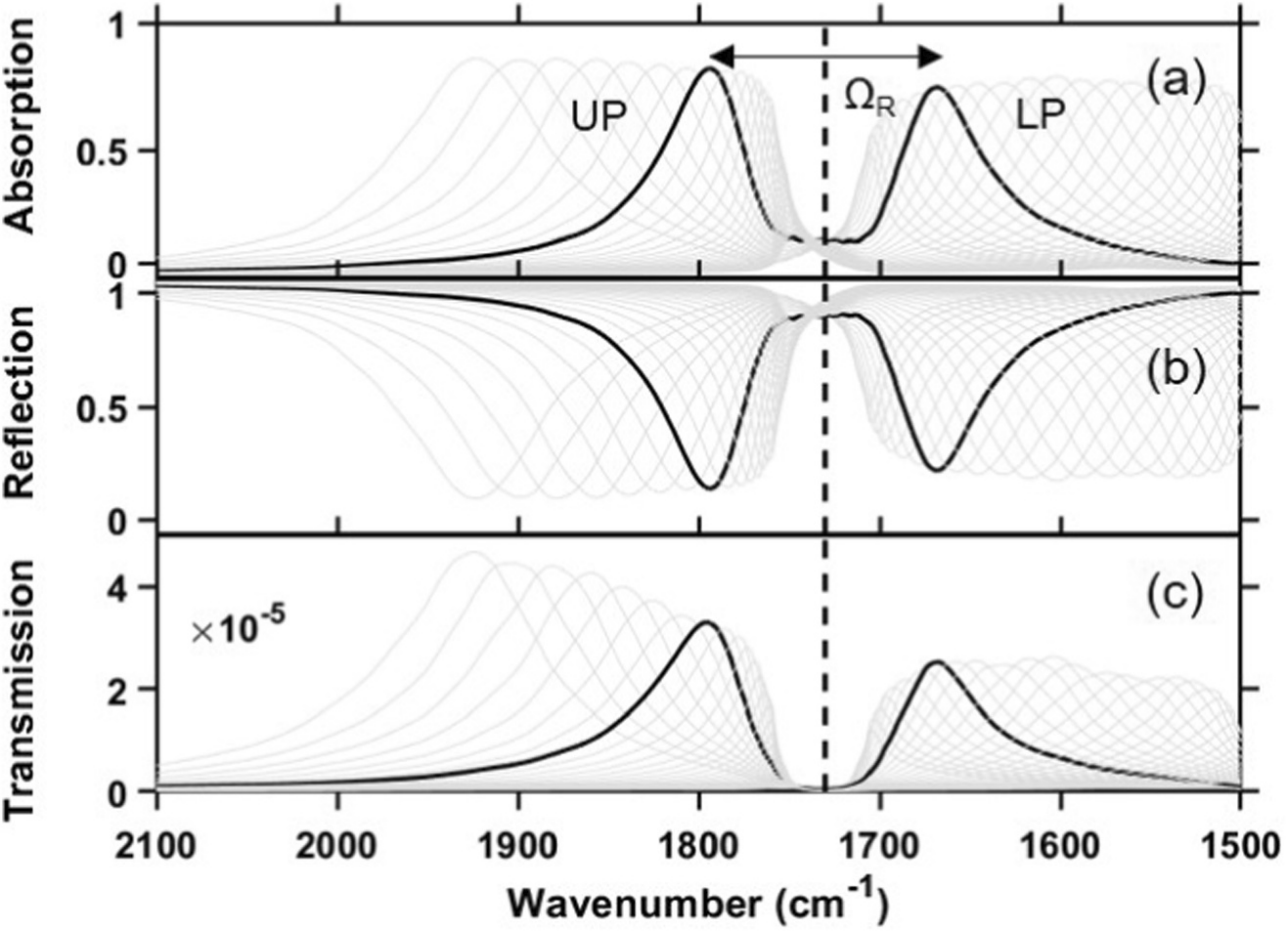
Simulated absorption, reflection, and transmission spectra of a PMMA‐integrated deep Ag grating with $t_{\text{top}}$ of 55 nm, shown for varying *h* from 0.5 to 0.9 μm (gray lines). The spectra at *h* = 0.67 μm are highlighted in black.

However, when $t_{\text{top}}$ is further increased, the absorption spectra begin to show a distinct peak at the molecular resonance, which is associated with the formation of *uncoupled* states. Figure 3 shows the *h*‐dependent absorption intensity maps and corresponding spectra of the PMMA‐integrated deep Ag grating with $t_{\text{top}}$ of (a) 144 nm, (b) 610 nm, and (c) 2584 nm. In all three cases, the gratings exhibit clear spectral splitting, with $\Omega_{R}$ of (a) 126.9 cm^−1^, (b) 128.0 cm^−1^, and (c) 128.6 cm^−1^, satisfying the strong coupling criterion for all $t_{\text{top}}$. However, as $t_{\text{top}}$ increases to 610 nm in (b), an additional *h*‐*independent* absorption peak emerges at 1730 cm^−1^, which is absent for the grating with a thinner PMMA layer of 144 nm in (a). This feature appears as an extra peak between the UP and LP branches, and its intensity increases further as $t_{\text{top}}$ is increased to 2584 nm in (c). In the absorption intensity maps, this *h*‐independent resonance manifests as a horizontal line at 1730 cm^−1^, whose intensity increases with $t_{\text{top}}$. In Supporting Information S1: Figure S5, the absorption and reflection spectra exhibit a feature at 1730 cm^−1^ as a peak and dip, respectively, whereas this feature is absent in the transmission spectra even with a high $t_{\text{top}}$ of 2584 nm. This is because the molecular resonance only weakly perturbs the net transmitted intensity after multiple interferences within the thick metal grating, while it produces a more pronounced change in the reflected field and in the absorbed power. Consequently, absorption and reflection are more suitable probes for revealing dark and uncoupled states.

**FIGURE 3:**
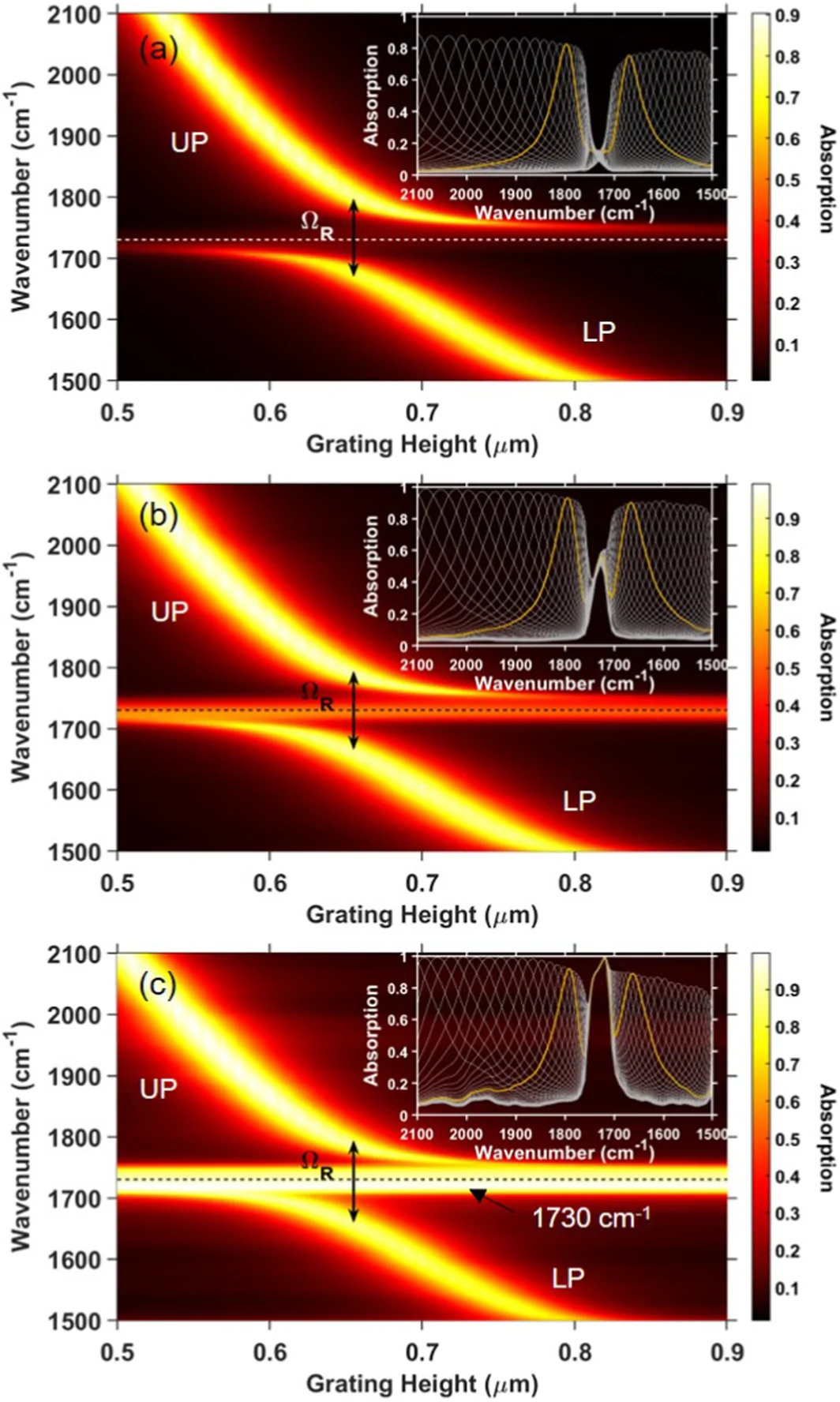
The simulated *h*‐dependent absorption intensity maps of PMMA‐integrated deep Ag gratings with $t_{\text{top}}$ of (a) 144 nm, (b) 610 nm, and (c) 2584 nm. The insets show absorption spectra as a function of *h* (white lines) and at *h* = 0.66 μm for (a) and 0.65 μm for (b) and (c) (orange lines). Horizontal dotted lines indicate the PMMA carbonyl stretching mode at 1730 cm^−1^.

Spectral features appearing near the molecular resonance frequency have been interpreted differently across various light–matter coupled systems. For instance, a transmission peak emerging between the UP and LP branches was attributed to plasmon‐mediated dipole‐dipole coupling in a Ag slit array incorporating a molecular layer [30]. In another study, a reflectivity dip was assigned to uncoupled intersubband absorption in semiconductor quantum wells coupled to an inductance‐capacitance resonant circuit [72]. When the quasi‐bound‐state‐in‐the‐continuum (q‐BIC) resonance of the Ge metasurface is strongly coupled to the C=O vibrational resonance of PMMA, a small transmission dip remained at the bare C=O vibrational frequency [73]. This residual dip was attributed to absorption by PMMA located directly on the substrate, where the molecules lie outside the region of effective q‐BIC mode overlap. Theoretically, additional peaks observed in organic microcavities have been explained in terms of dark vibronic polaritons [23, 24], or more generally as dark polaritons [25]. As such, spectral features centered around the bare molecular resonance are often loosely attributed either to dark states [74] or to uncoupled molecules [75, 76], despite the fact that these represent fundamentally distinct physical entities with different origins and properties. Dark states are intrinsic to the coupled system and arise from collective quantum superpositions of N molecules within the cavity mode volume (i.e., they are coupled but optically inactive). In contrast, uncoupled states originate from molecules located outside the effective mode volume and are therefore truly uncoupled from the cavity field [21]. While dark states exist for each cavity mode and are confined within regions of strong cavity field, the population of uncoupled states instead scales with the bulk volume of excess molecules [19].

To corroborate each manifold of polaritonic, dark, and uncoupled states, we simulated the *E*‐field intensity distributions for PMMA‐integrated deep Ag gratings with $t_{\text{top}}$ of (a) 144 nm, (b) 610 nm, and (c) 2584 nm at the (i) LP, (ii) molecular resonance (1730 cm^−1^), and (iii) UP frequencies, as shown in Figure 4. For each case, we also plot the *E*‐field intensity profiles along the *z*‐axis at the center of a trench gap (dashed vertical lines), highlighting the field at the grating—PMMA interface (colored lines). At all three frequencies, the PMMA‐integrated deep Ag grating exhibits strongly enhanced *E*‐fields confined within the trench gaps, particularly near the opening of the Ag grating. These amplified *E*‐fields decay along the *z*‐axis toward the bottom of the PMMA‐filled trench and into the top PMMA overlayer, forming exponentially‐decaying evanescent waves associated with the grating mode. The characteristic decay lengths of the grating field into the top PMMA layer ($\delta_{\text{grating}}$), calculated by a factor of 1/e to the maximum electric field intensity, lie in the range of 43 to 45 nm (Supporting Information S1: Table S1). In addition, the enhanced fields propagate into the bulk Ag, generating periodic *E*‐field patterns along the *x*‐direction.

**FIGURE 4:**
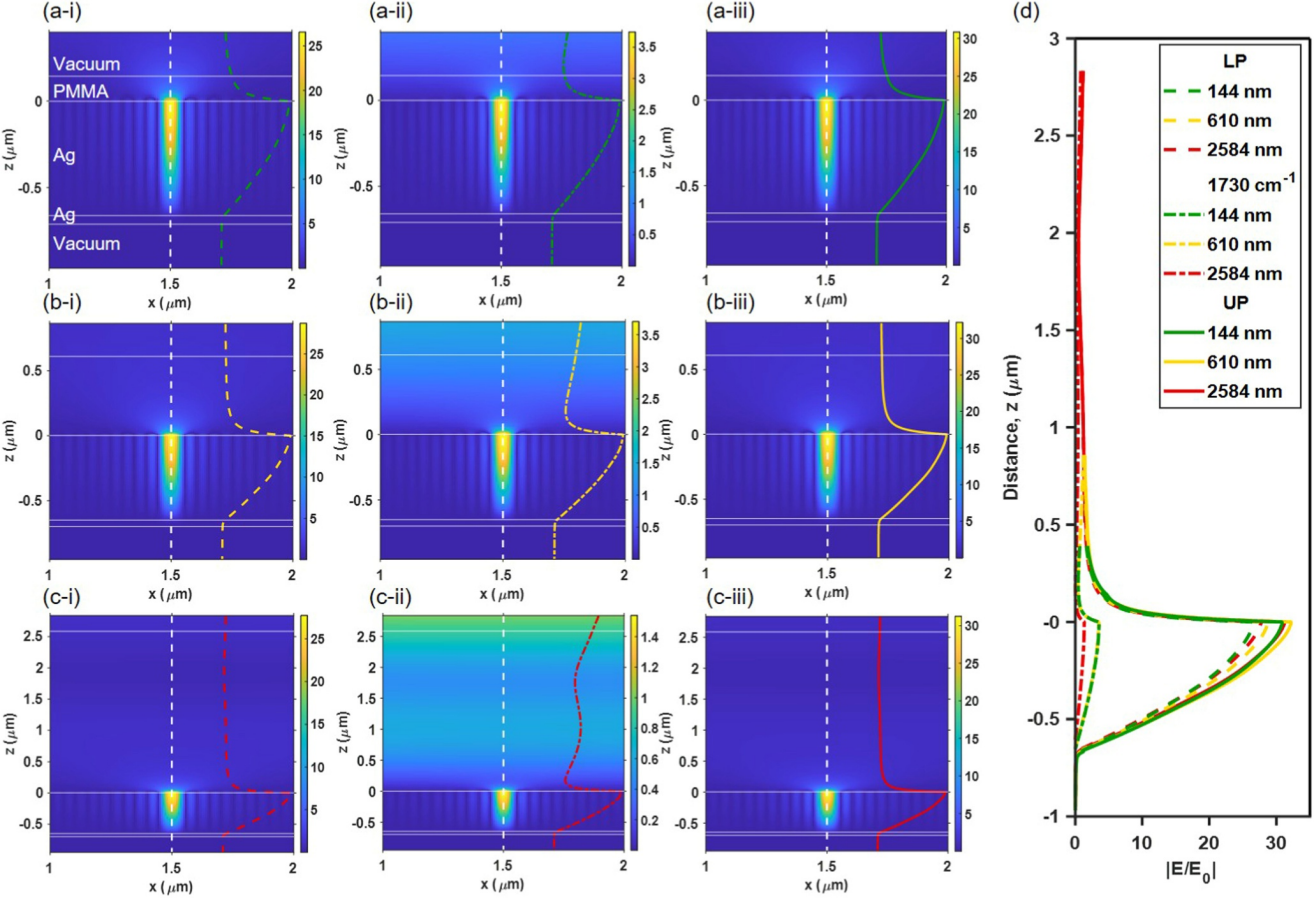
RCWA‐simulated *E*‐field intensity maps of PMMA‐integrated deep Ag gratings with $t_{\text{top}}$ of (a) 144 nm, (b) 610 nm, and (c) 2584 nm at the (i) LP, (ii) 1730 cm^−1^, and (iii) UP frequencies. *E*‐field intensity profiles extracted from the center of the trench gap (indicated by dashed vertical lines) are plotted as colored lines in (a–c). A comparison of all *E*‐field intensity profiles is shown in (d).

Although the spatial *E*‐field profiles appear qualitatively similar for gratings with a different $t_{\text{top}}$ in Figure 4a–c, the absolute *E*‐field amplitudes at the grating—PMMA interface differ significantly between frequencies (Figure 4d). The *E*‐field intensity is enhanced by factors of 8.2, 8.7, and 20 at the UP and LP frequencies relative to the molecular resonance frequency for $t_{\text{top}}$ of 144, 610, and 2584 nm, respectively. The comparatively low *E*‐field intensity at 1730 cm^−1^ seems inconsistent with the strong absorption observed at the molecular resonance, particularly for a thick PMMA layer shown in Figure 3c. However, the high absorption despite significantly reduced *E*‐field at 1730 cm^−1^, in fact, confirms that there is a large uncoupled molecular population that maintains its intrinsic absorption characteristics independent of local field enhancement. The uncoupled molecules absorb at the bare C=O frequency of 1730 cm^−1^, but they do not participate in the collective coupling, and therefore do not benefit from the enhanced *E*‐fields that create the polaritonic states. In contrast, polaritonic states arise from collective coupling, where coherent interactions among many molecules simultaneously amplify both field enhancement and absorption. This contrast is most clearly observed in the grating with a thick PMMA layer, showing the strongest absorption at 1730 cm^−1^ in Figure 3c, but the weakest *E*‐field intensity at the same frequency for $t_{\text{top}}$ = 2584 nm in Figure 4d (dashed‐dot lines). Therefore, as $t_{\text{top}}$ increases, uncoupled states dominate the molecular population at the bare vibrational frequency.

### 2.3 VSC‐Induced Self‐Stratification of a Polymer Film

Figure 5 shows the complete trajectories of (a) $\Omega_{R}$ and (b) $A^{{1730\text{cm}}^{-1}}$, each versus $\sqrt{t_{\text{top}}}$. The plots exhibit three distinct regions, (I) through (III), indicating that different underlying mechanisms dominate in each region. In region (I), $\Omega_{R}$ increases with $\sqrt{t_{\text{top}}}$, following the linear $\sqrt{N}$ dependence of the collective coupling strength for molecules located within $t_{\text{top}}$ < 377 nm ($\sqrt{t_{\text{top}}}$ of 19.4 nm^1/2^ in Figure 5a). This behavior is analogous to thin excitonic films showing standard $\sqrt{N}$ scaling when coupled to surface plasmon polariton modes [77]. In this region, most C=O oscillations hybridize into polaritonic states, leaving $A^{{1730\text{cm}}^{-1}}$ at a low but non‐zero constant value. The dark states present in region (I) correspond to an approximately 15%–26% baseline in $A^{{1730\text{cm}}^{-1}}$ for $t_{\text{top}}$ between 0 and 377 nm (Figure 5b). However, the contribution of uncoupled states remains negligible in region (I), because $t_{\text{top}}$ is still sufficiently thin and confined close to the evanescent field of the grating mode.

**FIGURE 5:**
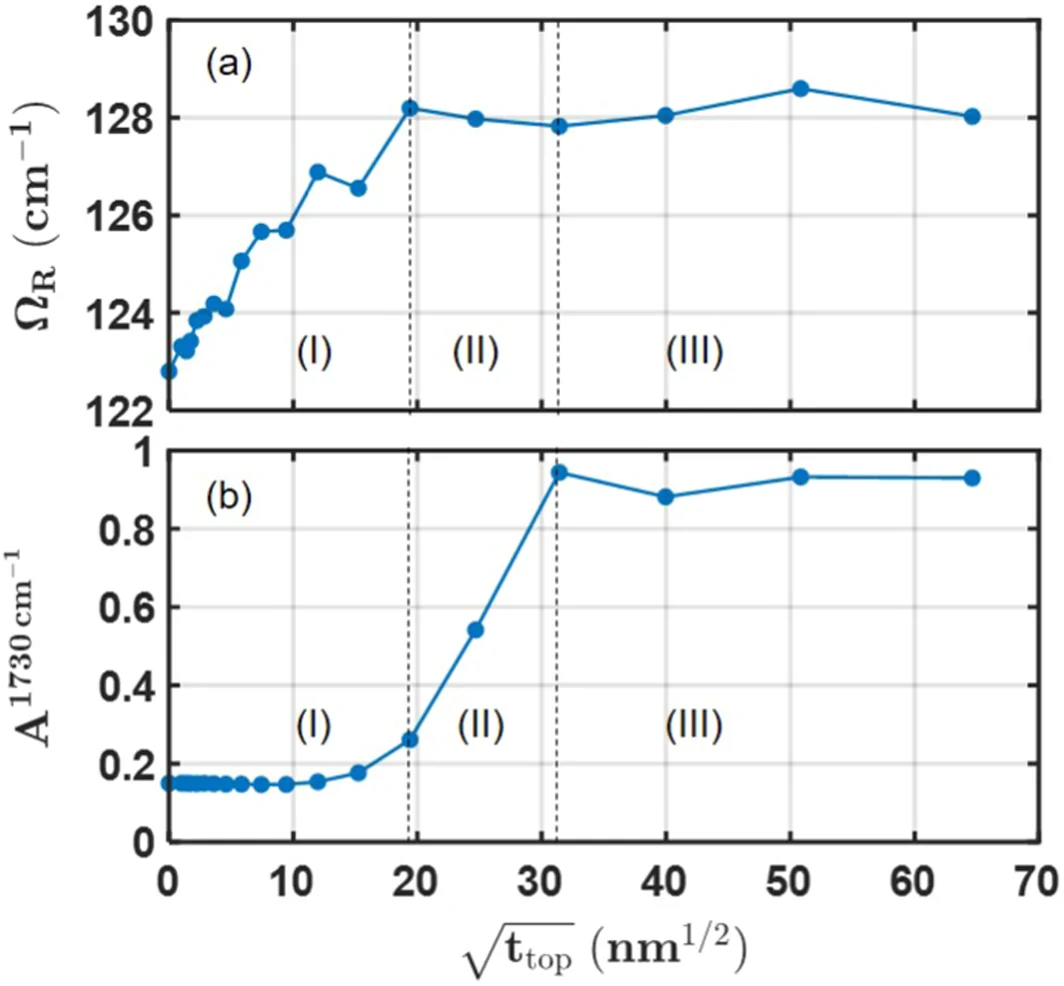
Dependence of the Rabi splitting ($\Omega_{R}$) and the absorption intensity at 1730 cm^−1^ ($A^{{1730\text{cm}}^{-1}}$) on $\sqrt{t_{\text{top}}}$. As $t_{\text{top}}$ is increased from 0 to 4181 nm, (a) $\Omega_{R}$ and (b) $A^{{1730\text{cm}}^{-1}}$ reveal three distinct regions of (I), (II), and (III), corresponding to a transition from polaritonic and dark states to uncoupled bulk‐like response.

Region (II) shows the transition from polaritonic—dark dominated states to a regime where uncoupled states become increasingly important, characterized by $\Omega_{R}$ reaching a maximum and $A^{{1730\text{cm}}^{-1}}$ rising sharply for 377 nm < $t_{\text{top}}$ < 987 nm. In this region, the mode volume becomes saturated, so additional PMMA lies beyond the effective decay length of the grating mode. The extra molecules then would not increase the effective N in the strong coupling, so $\Omega_{R}$ stops growing. Nonetheless, these extra molecules still absorb at the bare PMMA C=O frequency (1730 cm^−1^), increasing $A^{{1730\text{cm}}^{-1}}$ up to $t_{\text{top}}$ of 987 nm.

As $t_{\text{top}}$ increases further beyond the mode confinement region, the excess PMMA acts as a bulk material, absorbing at the original C=O vibration frequency between the UP and LP branches. In region (III), both $\Omega_{R}$ and $A^{{1730\text{cm}}^{-1}}$ saturate, resulting in the deviation of $\Omega_{R}$ from the standard $\sqrt{N}$ scaling. Similar saturation of the effective coupling strength has been reported in systems where emitters interact with propagating evanescent optical modes, such as strongly coupled CdSe nanoplatelets with plasmonic hole arrays [78] and dye molecules coupled to lattice modes of dielectric nanoparticle arrays [79].

Therefore, in collective VSC, $\Omega_{R}$ probes the fraction of PMMA molecules that overlaps with the confined grating mode, rather than the total amount of polymer through an intensity change at a selected frequency. $\Omega_{R}$ directly quantifies the formation and evolution of the hybrid polaritonic states and can serve as a sensing parameter for determining whether a perturbation has reached the strongly coupled region. The boundaries in Figure 5, however, indicate approximate crossover thicknesses rather than abrupt transitions. At low PMMA thicknesses, the increasing field‐overlapping molecular population enhances the collective coupling strength. As the PMMA layer becomes thicker, an increasing fraction of the added polymer lies outside the high‐field region. The Rabi splitting consequently approaches saturation, whereas the absorption near the bare C=O vibration progressively reflects contributions from weakly coupled and effectively uncoupled PMMA.

Our results demonstrate that strong coupling between grating modes and molecular vibrations can drive an effective self‐stratification of a polymer film. Despite being chemically identical, PMMA molecules within $t_{\text{top}}$ of 377 nm exhibit the properties of vibrational polaritons, whereas PMMA molecules located above $t_{\text{top}}$ of 377 nm retain the bulk‐like properties of uncoupled PMMA. The cavity‐coupled PMMA shows dispersive UP and LP branches, while the uncoupled bulk PMMA exhibits a flat vibrational mode that produces the strong absorption peak. Therefore, the system behaves as an effective bilayer, in which the lower layer is modified by strong coupling within the evanescent field of the deep Ag grating, and the upper layer remains optically equivalent to bare PMMA.

### 2.4 Solvent‐Diffusion Sensing

Utilizing the binary response of a polymer film formed by VSC, we now demonstrate solvent‐diffusion sensing. The grating is initially integrated with a uniform 1600 nm‐thick PMMA film, as shown in Figure 6a. Upon wetting the top surface of the PMMA layer with ethanol, the polymer is expected to swell [80], accompanied by changes in its optical constants. These changes are modeled using a linear mixing approximation between PMMA and ethanol: $n_{\text{swollen}}=\phi_{\text{PMMA}}n_{\text{PMMA}}+\phi_{\text{ethanol}}n_{\text{ethanol}}$, where $n_{\text{swollen}}$ is the effective refractive index of the swollen PMMA layer, and n and ϕ are the refractive index and volume fraction, respectively, of PMMA and ethanol (Supporting Information S1: Figure S6). Note that this treatment does not imply a reduction in the total number of C=O groups in the polymer. Assuming 30% swelling of PMMA by ethanol yields $\phi_{\text{PMMA}}$ and $\phi_{\text{ethanol}}$ of 0.77 and 0.23, respectively. While conventional plasmonic sensing schemes typically rely on spectral shifts of surface plasmon resonance modes [81, 82], a VSC‐based open cavity is based on the evolution of $\Omega_{R}$ and $A^{{1730\text{cm}}^{-1}}$ for probing the depth profile of a diffusion process via self‐stratification within a polymer film.

**FIGURE 6:**
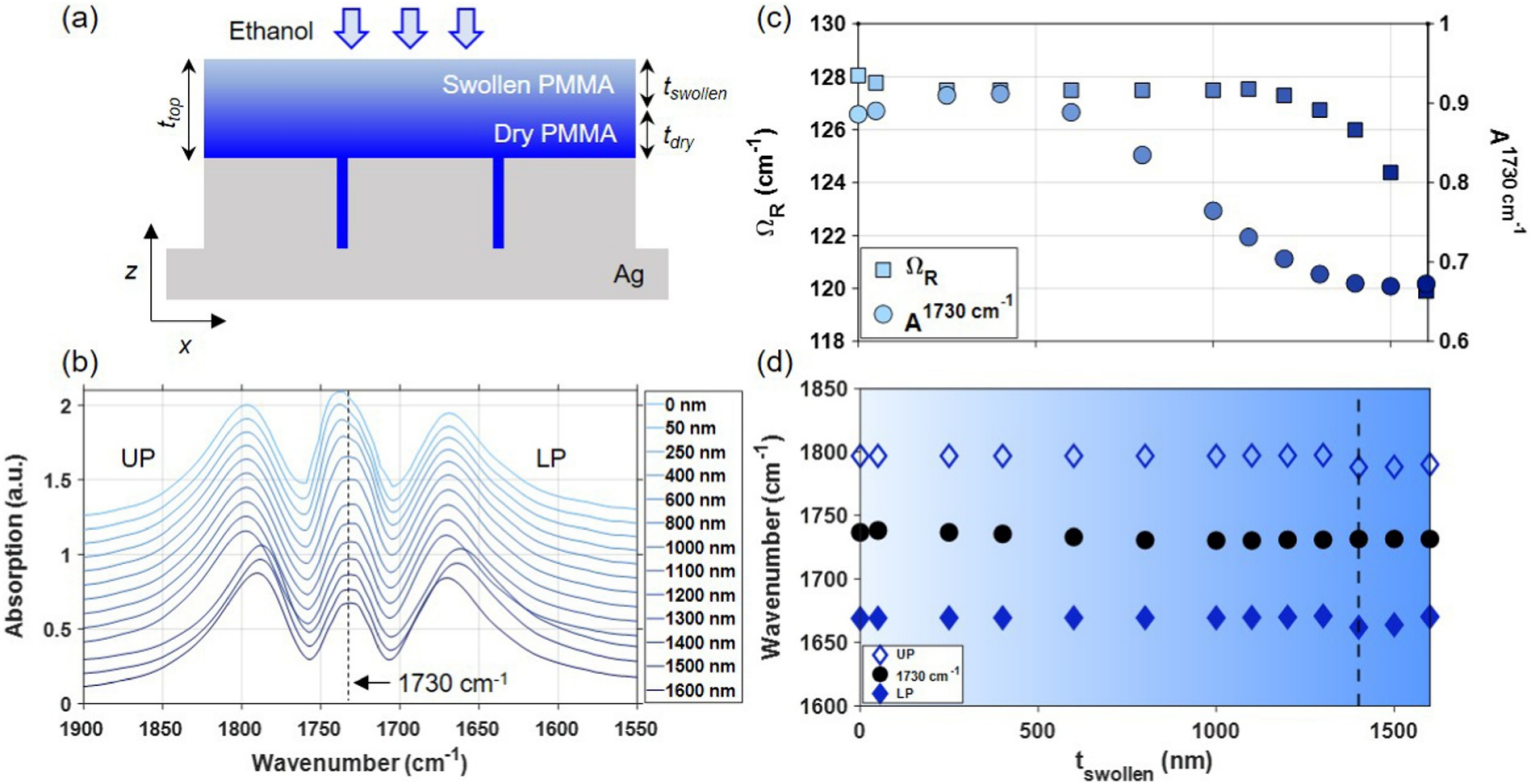
Demonstration of solvent‐diffusion sensing via VSC‐induced self‐stratification of a polymer film. (a) A schematic diagram of a PMMA‐integrated deep Ag grating with $t_{\text{top}}$ of 1600 nm. Upon addition of ethanol from the top surface, the PMMA layer separates into a swollen region of thickness $t_{\text{swollen}}$ and an underlying dry region of thickness $t_{\text{dry}}$. (b) Simulated absorption spectra for increasing $t_{\text{swollen}}$ from 0 (light blue) to 1600 nm (dark blue). (c) $\Omega_{R}$ (left *y*‐axis) and $A^{{1730\text{cm}}^{-1}}$ (right *y*‐axis) as a function of $t_{\text{swollen}}$ from 0 to 1600 nm. (d) Evolution of the UP branch, molecular peak at 1730 cm^−1^, and the LP branch with increasing $t_{\text{swollen}}$.

The evolution of the absorption spectrum is monitored as ethanol diffuses from the top into the dry PMMA layer over a distance, $t_{\text{swollen}}$ (Figure 6b). The UP and LP branches persist across the entire range of $t_{\text{swollen}}$, indicating that strong coupling is maintained at all thicknesses. For $t_{\text{swollen}}$ from 0 to 1200 nm, $\Omega_{R}$ remains nearly constant at 127.5 cm^−1^, as the swollen layer lies outside the mode volume and the effective number of coupled molecules remains unchanged (left *y*‐axis, Figure 6c). When $t_{\text{swollen}}$ > 1200 nm, $\Omega_{R}$ begins to decrease, reaching 119.9 cm^−1^ at 1600 nm. This reduction indicates that the swollen layer, characterized by a lower refractive index and diluted C=O oscillator strength (Supporting Information S1: Figure S6), has become sufficiently thick to enter the mode volume of the grating.

The evolution of $A^{{1730\text{cm}}^{-1}}$ also provides a complementary and independent readout for the solvent‐diffusion sensing (right *y*‐axis, Figure 6c). After remaining nearly constant up to $t_{\text{swollen}}$ of 600 nm, $A^{{1730\text{cm}}^{-1}}$ decreases in the range 600 nm < $t_{\text{swollen}}$ < 1200 nm, corresponding to region (II) in Figure 5b, where a transition from the polaritonic—dark states to uncoupled states occurs. The $A^{{1730\text{cm}}^{-1}}$ value then reaches a minimum value of 0.67 for $t_{\text{swollen}}$ = 1400–1600 nm, when the swollen layer reaches the strongly coupled interface. The decrease in $A^{{1730\text{cm}}^{-1}}$ with increasing $t_{\text{swollen}}$ again reflects the reduced oscillator strength of the swollen film. Therefore, the arrival of the solvent front at the coupled interface can be identified from the onset of the decrease in $\Omega_{R}$ and the evolution of $A^{{1730\text{cm}}^{-1}}$. Supporting Information S1: Figure S7 represents the corresponding absorption intensity maps of the deep Ag grating for varying $t_{\text{swollen}}$.

The spatial separation of the sensing regimes is further reflected by the frequency shifts of the polaritonic modes as the swollen PMMA front enters the strongly coupled region. Figure 6d shows that both the UP and LP branches exhibit an abrupt red shift at $t_{\text{swollen}}\geq$ 1400 nm, while the peak position of the C=O stretching mode remains unchanged with $t_{\text{swollen}}$. This behavior can be rationalized as follows: when swollen PMMA replaces dry PMMA within the mode volume, the reduced effective refractive index $n_{\text{swollen}}$ causes the bare cavity mode to shift toward the molecular resonance. As a result, both LP and UP branches shift to lower wavenumbers, with the UP approaching and the LP receding from the C=O vibrational mode at 1730 cm^−1^, accompanied by a reduction in $\Omega_{R}$. Distinct optical regions formed by VSC induce an effective optical self‐stratification of a polymer, and the evolution of the $\Omega_{R}$ and $A^{{1730\text{cm}}^{-1}}$ clearly demonstrates depth‐resolved solvent‐diffusion sensing. This behavior is fundamentally different from existing plasmonic sensors, which provide either surface‐sensitive or thickness‐averaged responses [83].

We emphasize that the capability of the deep Ag grating may go beyond solvent‐diffusion sensing. In principle, the same self‐stratification and field‐confinement concepts could be exploited to realize self‐filtering optical elements, in which only molecules residing within the strongly coupled region experience modified spectral responses, while the bulk‐like overlayer preserves its native absorption or emission characteristics. This built‐in selectivity in depth could be used to gate signals from specific layers or to suppress background from uncoupled molecules. Moreover, the sharp spatial confinement of the hybrid modes provides a natural platform for site‐selective chemistry or lithography. Reactions or patterning steps are activated preferentially within the near‐field region of the grating, leaving chemically identical material outside the mode volume largely unperturbed. Finally, the gradient in coupling strength and local density of optical states can be engineered to promote directional energy funneling within polymer or molecular films, guiding excitations from weakly coupled outer regions toward strongly coupled interfaces, with potential implications for light harvesting, signal routing, and polariton‐assisted transport.

## 3 Conclusion

We have demonstrated that strong coupling between deep Ag grating modes and the molecular vibrations of a polymer drives an effective self‐stratification within an otherwise homogeneous polymer film, producing a binary optical response that separates cavity‐coupled and bulk‐like regions. By combining *h*‐dependent absorption spectra with detailed near‐field analysis, we distinguished polaritonic, dark, and uncoupled manifolds and showed how their relative populations evolve with PMMA thickness, ultimately leading to saturation of the coupling strength once the grating mode volume is filled. This stratified optical landscape enabled a VSC‐based open cavity to function as a solvent‐diffusion sensor, where ethanol‐induced swelling of PMMA modulates the $\Omega_{R}$ and $A^{{1730\text{cm}}^{-1}}$, allowing us to track the arrival of the solvent front at the strongly coupled interface. Our results establish deep metal gratings integrated with molecular films as versatile platforms in which field confinement and evanescent mode profiles can be used to sculpt effective refractive index and absorption profiles, opening new pathways toward self‐filtering photonic elements, site‐selective chemistry and lithography, and controlled energy funneling in polymer and molecular systems.

## 4 Methods

The optical response of the deep Ag grating structures was calculated using rigorous coupled‐wave analysis (RCWA) implemented through a customized version of the Periodically Patterned Multi Layer (PPML) [84]. The PPML employs a scattering‐matrix‐based RCWA framework for the analysis of multilayer periodic structures with an infinitely repeated lateral unit cell. The original code was adapted to the specific grating geometries investigated in this work by modifying the layer definitions, implementing custom material distributions, and incorporating wavelength‐dependent optical constants for all materials considered. The complex refractive index data of Ag and PMMA were obtained from Yang, et al., [85] and Zhang, et al., [86, 87] respectively.

A periodic deep Ag grating was modeled on top of a 50‐nm‐thick bottom Ag film. The grating height was swept between 0.5 and 1.1 μm, while the gap width was chosen as 50 nm based on the highest efficiencies obtained from preliminary results. Depending on the investigated configuration, PMMA was introduced either inside the grating gaps, as a top overlayer, or simultaneously in both regions, resulting in three distinct grating structures. The region beneath the metal stack was treated as a semi‐infinite vacuum, whereas the superstrate was assumed to be either PMMA or vacuum depending on the configuration. The surrounding medium was therefore modeled with a relative permittivity of ε_r_ = 1 for vacuum. Plane‐wave illumination was assumed under near‐normal incidence, and both the reflected and transmitted diffraction orders were calculated. Finally, the wavelength‐dependent reflection and transmission were obtained from which absorption was calculated via energy conservation, *A* = 1 − *T* − *R*, with *T* ≈ 0 when the metal back‐reflector is optically opaque. The resulting absorption spectra were analyzed to identify cavity resonances, vibrational polariton formation, and the evolution of strong light–matter coupling as a function of cavity geometry and PMMA distribution.

## Author Contributions

**Yunus Cayir:** methodology, software, data curation, investigation, formal analysis, visualization, writing – review and editing. **Md Faysal Hossain:** methodology, software. **Wonmi Ahn:** conceptualization, methodology, software, data curation, investigation, validation, formal analysis, supervision, funding acquisition, visualization, project administration, resources, writing – original draft, writing – review and editing.

## Conflicts of Interest

The authors declare no conflicts of interest.

## Supporting information


Supporting Information S1

## Data Availability

The data that support the findings of this study are available from the corresponding author upon reasonable request.
