# Supplementary material for: Self‐Stratification of Polymer Films by Strong Coupling Between Molecular Absorbers and Infrared Grating Modes
Source: Nanophotonics. 2026 Nov 6;15(21):e70354. doi: 10.1002/nap2.70354 (PMC13741293; doi:10.1002/nap2.70354)
Supplement: Supplementary file 1 [file NAP2-15-e70354-s001.docx]

**Self-Stratification of Polymer Films by Strong Coupling between Molecular Absorbers and Infrared Grating Modes**

Yunus Çayır, Md Faysal Hossain, and Wonmi Ahn*

UNAM – National Nanotechnology Research Center and Institute of Materials Science and Nanotechnology, Bilkent University, Ankara, Türkiye

*E-mail: wonmi.ahn@unam.bilkent.edu.tr

**Supporting Information**

**Table S1.** Decay lengths of the grating field into the top PMMA layer (in nm).

**Figure S1.** RCWA-simulated absorption intensity maps of a bare deep Ag grating with varied trench width (*b*).

**Figure S2.** Absorption intensity maps of a deep Ag grating integrated with a PMMA layer on top of the grating, with the trench gaps left empty.

**Figure S3.** Absorption intensity maps of a deep Ag grating partially filled with PMMA, without a top PMMA layer.

**Figure S4.** Absorption intensity map of a deep Ag grating fully filled with PMMA, without a top PMMA layer.

**Figure S5.** Absorption, reflection, and transmission spectra of a PMMA-integrated deep Ag grating with $t_{top}$ of 2584 nm.

**Figure S6.** Changes in the refractive index (*n*) and extinction coefficient (*k*) of a PMMA layer swollen by ethanol.

**Figure S7.** Absorption intensity maps of a deep Ag grating containing a swollen PMMA film.

**Table S1.** Decay lengths of the grating field into the top PMMA layer (in nm).

| $t_{top}$ | At LP frequency | 1730 cm^-1^ | At UP frequency |
| --- | --- | --- | --- |
| 144 nm | 43.35 | 45.52 nm | 43.10 |
| 610 nm | 43.38 | 45.32 nm | 43.30 |
| 2584 nm | 43.50 | 45.45 nm | 43.42 |

**Figure S1.** RCWA-simulated absorption intensity maps of a bare deep Ag grating with varied trench width (*b*). The structural parameters are *P* = 3 μm and a bottom Ag film thickness of 50 nm. In (a-i) through (d-i), *h* was varied from 0.5 to 1.1 μm, while in (a-ii) through (d-ii), *h* was fixed at 0.67 μm. The *b* values were varied from 50, 100, 150, to 200 nm.

**Figure S2.** Absorption intensity maps of a deep Ag grating integrated with a PMMA layer on top of the grating, with the trench gaps left empty. The thickness of the top PMMA layer ($t_{top}$) is varied as **(a)** 144 nm, **(b)** 610 nm, and **(c)** 2584 nm. The UP and LP branches are formed around the C=O vibrational resonance of PMMA at 1730 cm^-1^ (horizontal dashed lines), together with an *h*-independent absorption feature at the molecular resonance frequency. The inset figures show absorption spectra at varying *h* (white lines) and at fixed *h* values of **(a)** *h* = 0.93 μm, **(b)** 0.90 μm, and **(c)** 0.90 μm (orange lines). The dependence of **(d)** the Rabi splitting ($\Omega_{R}$) and **(e)** the absorption intensity at 1730 cm^-1^ ($A^{{1730cm}^{-1}}$) on $\sqrt{t_{top}}$. High $A^{{1730cm}^{-1}}$ values for $t_{top}$ < 233 nm (i.e., $\sqrt{t_{top}}$ = 15.3 nm^1/2^) in (**e**) are attributed to polaritonic states that are not fully resolved.

**Figure S3.** Absorption intensity maps of a deep Ag grating partially filled with PMMA, without a top PMMA layer. The PMMA thickness within the trench gaps, $t_{gap}$, is set to **(a)** 300 nm and **(b)** 600 nm. In **(b)**, the UP and LP branches are formed around the C=O vibrational resonance of PMMA at 1730 cm^-1^ (horizontal dashed lines). The inset figures show absorption spectra as a function of *h* (white lines) and at fixed *h* values of **(a)** *h* = 0.96 μm and **(b)** 0.79 μm (orange lines). **(c)** The Rabi splitting ($\Omega_{R}$) and **(d)** the absorption intensity at 1730 cm^-1^ ($A^{{1730cm}^{-1}}$) as a function of $t_{gap}$.

**Figure S4.** Absorption intensity map of a deep Ag grating fully filled with PMMA, without a top PMMA layer. The UP and LP branches are formed around the C=O vibrational resonance of PMMA at 1730 cm^-1^ (horizontal dashed lines), forming $\Omega_{R}$ at *h* = 0.69 μm. **(b)** Absorption, **(c)** reflection, and **(d)** transmission spectra shown for varying *h* from 0.5 to 0.9 μm (gray lines) and at *h* = 0.69 μm (highlighted in black).

**Figure S5.** Absorption, reflection, and transmission spectra of a PMMA-integrated deep Ag grating with $t_{top}$ of 2584 nm. The spectra are obtained at varied *h* from 0.5 to 0.9 μm (gray lines). The spectra at *h* = 0.65 μm are highlighted in black. Both the absorption and reflection spectra show a feature at 1730 cm^-1^, appearing as a peak in absorption and a corresponding dip in reflection. However, no such feature is visible in transmission, even at a large $t_{top}$ of 2584 nm. This arises because the molecular resonance only weakly modifies the overall transmitted intensity after multiple interferences in the thick metal grating, while it induces a much stronger perturbation in the reflected field and in the absorbed power.

**Figure S6.** Changes in the refractive index (*n*) and extinction coefficient (*k*) of a PMMA layer swollen by ethanol. When the top surface of the PMMA layer is wet by ethanol, the PMMA swells (assumed 30% swelling), and its optical constants are modified following a linear mixing rule between PMMA and ethanol: $n_{swollen}=\phi_{PMMA}n_{PMMA}+\phi_{ethanol}n_{ethanol}$, where $n_{swollen}$ is the effective refractive index of the swollen PMMA layer (red line), and $n$ and $\phi$ are the refractive index and volume fraction, respectively, of PMMA and ethanol. The optical constants of bare PMMA and ethanol are shown as black and blue lines, respectively.

**Figure S7.** Absorption intensity maps of a deep Ag grating containing a swollen PMMA film. The grating is initially integrated with a uniform 1600 nm-thick PMMA film. Upon adding ethanol from the top surface, the PMMA layer separates into a swollen region of thickness $t_{swollen}$ and an underlying dry region of thickness $t_{dry}$. The swollen PMMA thickness $t_{swollen}$ is set to **(a)** 50 nm, **(b)** 1200 nm, and **(c)** 1600 nm to monitor how the $\Omega_{R}$ and $A^{{1730cm}^{-1}}$ evolve as the PMMA layer changes from partially to fully swollen PMMA layers. The inset figures show absorption spectra as a function of *h* (white lines) and at fixed *h* values of **(a)** *h* = 0.65 μm, **(b)** 0.65 μm, and **(c)** 0.66 μm (orange lines).
